# Autonomic Dysfunction in Patients with Acute Infection with *Coxiella burnetii*

**DOI:** 10.3390/pathogens15010003

**Published:** 2025-12-19

**Authors:** Branislav Milovanović, Nikola Marković, Elizabeta Ristanović, Sonja Atanasievska Kujović, Nikoleta Đorđevski, Masa Petrovic, Milica Milošević, Sulin Bulatovic, Milovan Bojić

**Affiliations:** 1 Institute for Cardiovascular Diseases “Dedinje”, 11040 Belgrade, Serbia; 2 School of Medicine, University of Belgrade, 11000 Belgrade, Serbia; 3 Institute for Microbiology, Military Medical Academy, 11000 Belgrade, Serbia

**Keywords:** *Coxiella burnetii*, autonomic dysfunction, ME/CFS, heart rate variability, syncope

## Abstract

**Background:** *Coxiella burnetii* is a common zoonotic pathogen that can lead not only to acute or chronic Q fever but also to post-infectious syndromes, where autonomic nervous system (ANS) dysfunction has been suggested as a contributing mechanism. This study aimed to assess autonomic function in patients presenting with polymorphic symptoms, dysautonomia, or ME/CFS who had serological evidence of acute infection with *Coxiella* burnetii. **Methods:** A total of 156 participants were evaluated, including 100 seropositive patients and 56 matched controls. All subjects underwent standardized cardiovascular reflex tests (CART), beat-to-beat analysis of heart rate and blood pressure with baroreflex indices, 24 h Holter ECG with HRV assessment, and, in the *Coxiella* group, head-up tilt testing (HUTT). **Results:** A significantly higher prevalence of autonomic dysfunction was observed in the *Coxiella* group, predominantly affecting parasympathetic regulation, with abnormal CART scores, reduced LF power and baroreflex effectiveness, and a high rate of positive HUTT findings characterized by extreme blood pressure variability. Although long-term HRV measures did not differ significantly between groups, short-term indices consistently indicated ANS impairment. **Conclusions:** These findings suggest that *Coxiella burnetii* infection may trigger persistent autonomic dysfunction, potentially contributing to the development of ME/CFS and syncope in affected individuals. Further longitudinal studies are needed to clarify pathophysiological mechanisms and clinical implications.

## 1. Introduction

*Coxiella burnetii* is a small, pleomorphic coccobacillus with a Gram-negative cell wall and is recognized as one of the most common zoonotic pathogens worldwide [1,2]. It primarily infects animals including wildlife and livestock such as cattle, goats, and sheep, although human infection may also occur [1,2]. Transmission to humans is most frequently associated with: (1) inhalation of aerosolized particles, (2) ingestion of raw milk or unpasteurized goat cheese, (3) transfusion of contaminated blood products, (4) vertical transmission, and (5) tick bites [3]. The route of exposure influences the clinical presentation, which may include pneumonia, influenza-like symptoms, or hepatitis [3].

In humans, infection is asymptomatic or subclinical in approximately 60% of cases. However, 4–5% of individuals progress to persistent localized infection, most commonly chronic Q fever, in which culture-negative endocarditis is the typical manifestation [1,2].

Diagnosis of acute or chronic *C. burnetii* infection relies primarily on serologic testing [2]. Infection triggers an antibody response to phase I and phase II antigens, with seroconversion typically occurring 7–15 days after symptom onset; by the third week, approximately 90% of patients have detectable antibodies [2]. The serologic gold standard is a fourfold rise in IgG and/or IgM titers against phase II antigens over a 3–6-week interval [1,2]. In primary infection, a phase II IgG titer ≥ 200 IU/mL and/or an IgM titer ≥ 50 IU/mL is considered diagnostically significant [2].

Although acute and chronic presentations of Q fever are well described, less common manifestations—including pericarditis, myocarditis, and pancreatitis—have also been reported [1]. Neurological complications are considered rare [4]. In a study of 1269 patients, neurological abnormalities were observed in 2.2% of cases, while among a subgroup of 121 patients, 40.5% reported headaches and 4.1% experienced confusion [5,6].

Furthermore, approximately 12–20% of patients with clinically manifest acute Q fever develop prolonged fatigue or ME/CFS-like symptoms, as described in previous studies [1,7]. Although 65–75% of ME/CFS cases follow a flu-like illness, increasing evidence suggests that autonomic nervous system (ANS) dysfunction plays a central role in both the onset and persistence of the syndrome [8,9,10,11]. The association between dysautonomia and infectious diseases has become a prominent area of interest, particularly in the post-COVID-19 era [12]. Previous studies have linked ANS dysfunction to multiple pathogens including SARS-CoV-2, *Borrelia burgdorferi*, HSV, VZV, and Mycoplasma pneumoniae, as well as to other zoonotic agents such as *Brucella* spp., where subclinical cardiac involvement and autonomic abnormalities have been reported during acute infection [12,13,14,15,16,17,18,19,20].

The aim of this study was to evaluate autonomic nervous system function in patients presenting with polymorphic symptoms (fatigue, cognitive impairment, myalgia), ME/CFS-like features, or other indicators of dysautonomia (syncope, orthostatic hypotension/intolerance, Postural Orthostatic Tachycardia Syndrome, etc.) who also demonstrated positive phase II IgM antibodies to *Coxiella burnetii*.

## 2. Materials and Methods

### 2.1. Patient Enrollment

This cross-sectional study included 100 participants over the age of 18 who were evaluated at the Neurocardiology Laboratory of the Cardiology Clinic, Institute for Cardiovascular Diseases “Dedinje.” Patients were consecutively referred for evaluation because of polymorphic symptoms (e.g., fatigue, cognitive impairment, joint pain, myalgia, sleep disturbances, low-grade fever) and/or signs of autonomic dysfunction (such as syncope, orthostatic hypotension, or postural orthostatic tachycardia syndrome (POTS)), and clinical diagnoses were established according to current guidelines [21,22,23,24,25]. All participants underwent standardized autonomic nervous system testing during the initial clinical visit. Following this assessment, patients were referred to accredited national reference institutions for serologic testing, which was typically performed within 2–5 days as part of the same diagnostic workup. The inclusion criteria for the Coxiella group required the presence of symptoms together with positive phase II IgM antibodies against *Coxiella burnetii*. Serologic testing for *Coxiella burnetii* phase II IgM antibodies was performed using a commercial ELISA assay (EUROIMMUN, Lübeck, Germany). Results were expressed as ratio values, and samples with a ratio ≥ 1.1 were interpreted as positive, in accordance with the manufacturer’s instructions. A total of 100 participants fulfilled these criteria and were included in the Coxiella group, while the 56 participants served as the healthy control group. The control group was age- and sex-matched, with no statistically significant demographic differences compared to the Coxiella group (Table 1).

Exclusion criteria for the Coxiella group included: (1) chronic medical conditions (e.g., neurological, endocrine, cardiological, rheumatological, or autoimmune) that could potentially explain the reported symptoms; (2) neurodegenerative diseases associated with primary autonomic failure, including Pure Autonomic Failure, Multiple System Atrophy, or Parkinson’s disease; and (3) suspected cross-reactivity of IgM antibodies with antigens of microorganisms other than *Coxiella burnetii*. All patients underwent comprehensive diagnostic evaluations, frequently including repeated consultations with neurologists, rheumatologists, endocrinologists, and other specialists, to ensure rigorous exclusion of alternative diagnoses.

### 2.2. Ethnic Approval

The study was approved by the Ethics Committee of the Institute for Cardiovascular Diseases “Dedinje” (approval No. 7548, dated 13 December 2023) and conducted in accordance with the Declaration of Helsinki. It was supported by grant 451-03-68/2020-14/200156 from the Ministry of Education, Science and Technological Development of the Republic of Serbia, and by the COVANSA grant from the Science Fund of the Republic of Serbia.

### 2.3. Study Protocol

All participants underwent a comprehensive battery of diagnostic assessments to evaluate autonomic nervous system (ANS) function, including Ewing’s cardiovascular autonomic reflex tests (CART), short-term (5 min) beat-to-beat heart rate and blood pressure analysis, short-term heart rate variability (HRV), and 24 h Holter ECG monitoring with long-term HRV analysis. The Head-Up Tilt Test (HUTT) was performed exclusively in the Coxiella group.

#### 2.3.1. Cardiovascular Autonomic Reflex Tests (CART)

CART consisted of five standardized tests assessing both branches of the ANS: two sympathetic tests—Handgrip Test (HGT) and Orthostatic Hypotension (OH)—and three parasympathetic tests—Valsalva Maneuver (VM), heart rate response to deep breathing (HRB), and heart rate response to standing (HRS) [26]. Ewing’s original classification categorizes results as normal, borderline, or abnormal; however, based on the recommendations of Bellaver et al., borderline results were considered normal due to their potential association with technical variability [26,27]. Each test result was assigned a numerical value (normal = 0, borderline = 1, abnormal = 2), and the cumulative score ranged from 0 to 10. Sympathetic dysfunction was defined as at least one abnormal sympathetic test. Early parasympathetic dysfunction was indicated by one abnormal test, while two or more abnormal parasympathetic tests denoted definite parasympathetic dysfunction [26,27].

#### 2.3.2. Short-Term Beat-to-Beat Analysis

Beat-to-beat heart rate and blood pressure variability were recorded in the supine position using the Task Force Monitor, employing the vascular unloading method with automated calibration against oscillometric measurements on the contralateral arm [28]. Parameters obtained included heart rate (HR), systolic and diastolic blood pressure (SBP and DBP), and spectral HRV indices—power spectral density (PSD) and frequency band powers: very-low-frequency (VLF, 0–0.05 Hz), low-frequency (LF, 0.05–0.17 Hz), and high-frequency (HF, 0.17–0.40 Hz), expressed in both absolute units (ms^2^) and normalized units (nu). The LF/HF ratio was calculated as a marker of sympathovagal balance. Baroreflex sensitivity (BRS, ms/mmHg) and the Baroreflex Effectiveness Index (BEI, %) were calculated according to Parati’s methodology [29].

#### 2.3.3. 24 h Holter ECG Monitoring

A three-lead 24 h Holter ECG was analyzed by an experienced cardiologist. In addition to mean heart rate values, long-term HRV was assessed, including frequency-domain parameters described above and the following time-domain indices: SDNN (standard deviation of normal RR intervals), RMSSD (root mean square of successive differences), and pNN50 (percentage of successive RR intervals differing by >50 ms). HRV analysis was performed using commercially available software.

#### 2.3.4. Head-Up Tilt Test (HUTT)

HUTT was performed using the non-pharmacological Westminster protocol with continuous non-invasive monitoring of heart rate and blood pressure [30]. Patients rested supine for 10 min before being tilted to 70° for up to 30 min. The test was considered positive if syncope or severe presyncope occurred. In addition to the positive/negative outcome, hemodynamic patterns during the passive phase were recorded, including extreme blood pressure variations (EVBP), small blood pressure variations (SVBP), hypertensive or extreme hypertensive responses, and the presence of POTS.

### 2.4. Statistical Analysis

Data are presented as mean ± standard deviation (SD), median (Mdn) with interquartile range (IQR, 25–75%), or counts (percentage), depending on data type. Normality of continuous variables was assessed using the Smirnov test and by visual inspection of histograms and Q–Q plots.

Group comparisons were performed using parametric tests (Independent Samples *t* test) or nonparametric tests (Chi Square test, Fisher’s exact test, and Mann–Whitney U test) for categorical or non-normally distributed continuous data.

Statistical analyses were conducted using SPSS version 26.0. A two-sided *p* value < 0.05 was considered statistically significant.

## 3. Results

The demographic characteristics of the study population are presented in Table 1. As noted, there were no statistically significant differences between the Coxiella and control groups with respect to age or sex distribution. The majority of participants in both groups were female, with a mean age above 40 years. Within the Coxiella group, the most frequently observed clinical condition was ME/CFS, followed by a history of previous syncope. Other chronic comorbidities were present in fewer than 5% of participants.

**Figure 1:**
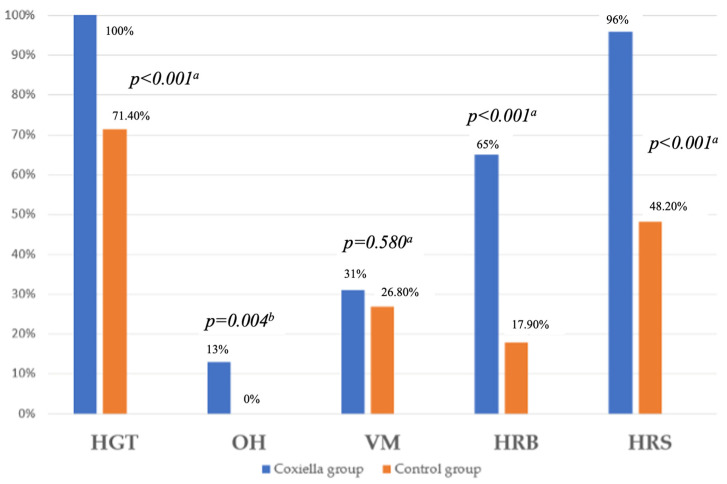
Abnormal results of Cardiovascular reflex tests of the study population. HGT—Hand grip test; OH—Orthostatic hypotension; VM—Valsalva maneuver; HRB—Heart rate response to deep breathing; HRS—Heart rate response to standing; ^a^—Pearson Chi Square; ^b^—Fisher Exact test.

Table 2 and Figure 1 summarize the results of the cardiovascular autonomic reflex tests (CART). Abnormal findings across all tests, except for the Valsalva maneuver, were significantly significant when comparing the Coxiella group compared with the control group. As expected, the overall autonomic neuropathy (AN) score was also substantially higher in the Coxiella group (*p* < 0.001).

**Figure 2:**
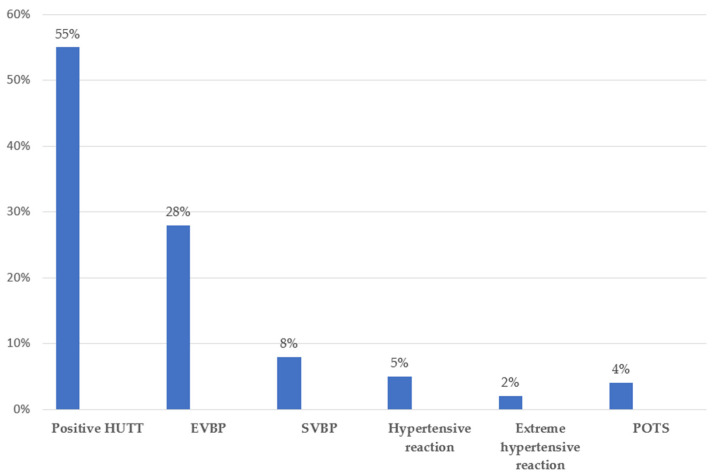
Outcomes of head up tilt test in Coxiella group. HUTT—Head up tilt test; EVBP—Extreme variations of blood pressure; SVBP—Small variations of blood pressure; POTS—Postural Orthostatic Tachycardia Syndrome.

Figure 2 presents the Head-Up Tilt Test (HUTT) outcomes in the Coxiella group. The test yielded positive results in more than 50% of participants. The most frequently observed hemodynamic abnormality was extreme blood pressure variability (EVBP), occurring in nearly 30% of the cohort. Postural Orthostatic Tachycardia Syndrome (POTS) was identified in approximately 4% of participants.

**Figure 3:**
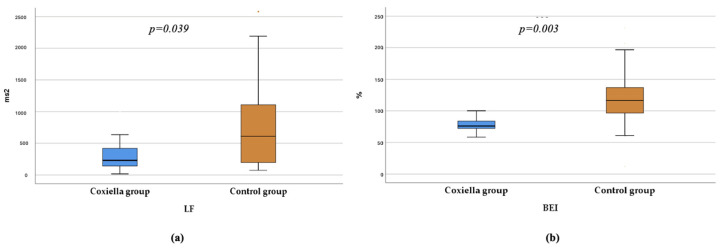
(**a**) Box plot of LF values in the Coxiella and control groups. (**b**) Box plot of BEI values in the Coxiella and control groups. LF—Low frequency component of HRV; BEI–Baroreflex effectiveness index; ms2—miliseconds squared; *p*-values correspond to the Mann–Whitney U test.

Table 3 presents the beat-to-beat analysis findings for the study population. Heart rate was significantly higher in the Coxiella group compared with controls (*p* = 0.015). Although all heart rate variability (HRV) parameters demonstrated lower median values in the Coxiella group, only the low-frequency (LF) component (expressed in ms^2^) and the Baroreflex Effectiveness Index (BEI) reached statistical significance when compared with the control group. Figure 3 presents the box plots for LF and BEI.

Table 4 summarizes the 24 h Holter ECG monitoring parameters for the study population. Although SDNN and most frequency-domain HRV indices demonstrated lower median values in the Coxiella group, none of these differences reached statistical significance. In both groups, LF/HF ratios exceeded a value of 2.

## 4. Discussion

The findings of this study indicate a presence of autonomic dysfunction, predominantly parasympathetic, in patients presenting with polymorphic complaints and/or symptoms of dysautonomia or ME/CFS who also exhibited positive serology testing for acute infection with *Coxiella burnetii*. This dysfunction was primarily demonstrated using Ewing’s CART.

As shown in Table 1, patients in the Coxiella group had a median age of approximately 40 years and were predominantly female. Although acute *C. burnetii* infection is typically reported more frequently in older individuals and in males, possibly due to the immunomodulatory effects of 17-β estradiol, our findings differ from this pattern [2,31,32]. This female predominance may reflect the high prevalence of ME/CFS (35%) and a history of syncope (59%) in the Coxiella group, both conditions known to affect women more often [33,34]. Previous studies have shown that 12–20% of individuals develop ME/CFS following clinically manifested acute Q fever [1,7]. In our cohort, the presence of parasympathetic dysfunction may contribute to the persistence of symptoms after *C. burnetii* exposure, given the established role of autonomic balance in modulating inflammatory responses [10,35,36]. Importantly, patients with ME/CFS in this study were not followed longitudinally from the onset of infection; rather, serology retrospectively confirmed prior exposure.

The high prevalence of prior syncope in our cohort (Table 1) is noteworthy, as syncope is a well-recognized clinical marker of autonomic dysfunction. Milovanović et al. previously reported that 58–63% of patients with vasovagal syncope or orthostatic hypotension (OH) had positive IgM antibodies to at least one infectious agent [37], linking a major manifestation of dysautonomia with the presence of infection. *Coxiella burnetii* was among the pathogens identified in that study [37]. In addition, numerous investigations have demonstrated autonomic abnormalities across a wide range of infectious diseases, an association that has become particularly evident in the post-COVID era [12,13,14,15,16,17,18,19].

As shown in Table 1, about 5% of our cohort had a Post COVID Syndrome (PCS) diagnosis. Co-infection with *C. burnetii* and SARS-CoV-2 has been reported [38], but current data do not suggest increased *C. burnetii* reactivation in PCS, unlike reactivations of EBV, CMV, or HHV-6 [39].

Table 2 shows that abnormal CART findings were more frequent in the Coxiella group (*p* < 0.05), except for the VM. Apparent sympathetic abnormalities in both groups were largely driven by the HGT, a test known to be unreliable because patients often unintentionally perform Valsalva during the maneuver [40,41]. Several authors have therefore questioned its value due to poor correlation with other Ewing battery components [42], and HGT responses are additionally affected by baseline diastolic pressure and hypertension [43].

In contrast, OH—an indicator of sympathetic dysfunction—was observed only in the Coxiella group, but in a relatively small proportion of patients (~15%) (Table 2, Figure 1). This is consistent with the findings of Milovanović et al., who reported that IgM positivity for *C. burnetii* was a negative predictor of OH during HUTT in univariable models [37], suggesting that marked sympathetic dysfunction is not a dominant feature of *Coxiella*-associated dysautonomia. Prior studies have shown that OH is far more common in late-stage Lyme disease than in ME/CFS or PCS, typically due to Small Fiber Neuropathy (SFN) [14,15]. Since *C. burnetii* has not been linked to SFN, the limited occurrence of OH in our cohort most likely reflects baroreflex impairment and milder degrees of sympathetic dysregulation rather than a primary sympathetic neuropathy.

Regarding parasympathetic dysfunction (Table 2), similar findings have been reported in ME/CFS and PCS, where CART and short-term HRV commonly show reduced vagal activity with compensatory sympathetic overdrive [15,44,45,46]. Proposed mechanisms include involvement of brainstem autonomic nuclei in neurotropic infections such as COVID-19 and *Borrelia* spp. [45,47]. Although overt CNS complications are rare in acute *C. burnetii* infection [5,6], impairment of peripheral or central reflex arcs—such as Bainbridge and baroreceptor pathways assessed by VM and HRS—remains possible [48,49]. Parasympathetic tone also decreases in systemic inflammation, diminishing vagal anti-inflammatory modulation and potentially contributing to dysautonomia [50]. These mechanisms may collectively explain the parasympathetic impairment observed in our cohort.

As shown in Figure 2, 55% of patients had a positive HUTT, which aligns with the high prevalence of prior syncope in this group (Table 1). The most common abnormality was extreme blood pressure variability, a pattern also reported in ME/CFS and PCS, where it appears in ~45% of patients with previous syncope [45]. Such fluctuations likely reflect impaired short-term baroreceptor regulation and are strong predictors of vasovagal responses during HUTT [51]. Only 4% of patients exhibited POTS, consistent with its higher prevalence in PCS and infections such as Mycoplasma or Borrelia, but not *Coxiella* [13,18,52]. Overall, these HUTT findings indicate underlying dysautonomia and may contribute to orthostatic intolerance, although further studies are needed to clarify the mechanisms.

As seen in Table 3, all short-term HRV parameters were lower in the Coxiella group, indicating reduced overall autonomic activity, although only LF (ms^2^) reached statistical significance (*p* = 0.039). While LF was traditionally viewed as a marker of sympathetic tone, later studies show that it reflects combined sympathetic–parasympathetic modulation and is strongly influenced by baroreflex function [53,54]. In parallel, the BEI-a direct measure of baroreflex responsiveness—was also significantly lower in the Coxiella group, pointing to impaired baroreceptor control [55]. Taken together with the CART and HUTT findings, the reductions in LF and BEI support the presence of baroreflex and parasympathetic dysfunction, which may contribute to orthostatic symptoms, blood pressure variability, and syncope observed in this cohort.

In contrast, 24 h Holter ECG monitoring showed no significant differences between groups (Table 4). This is expected, as 24 h HRV predominantly reflects sympathetic influences, whereas short-term recordings are more sensitive to parasympathetic changes [56]. A similar divergence-reduced short-term HRV with preserved 24 h values—has been reported in COVID-19 and PCS [45,56]. Comparable findings have also been described in other zoonoses, such as brucellosis, where altered LF/HF ratios and QT parameters suggest subclinical cardiac involvement and autonomic imbalance, underscoring the importance of arrhythmia surveillance in infectious diseases [20].

Taken together, the CART, HRV, and HUTT findings in our study show a consistent pattern of autonomic involvement in patients with acute *Coxiella burnetii* infection, characterized predominantly by reduced parasympathetic activity with accompanying baroreflex disturbances. This is reflected through abnormal heart-rate responses on CART, lower short-term HRV values, particularly LF and BEI, and a broad range of tilt-induced reactions, including pronounced blood pressure variability and orthostatic changes. Such a pattern aligns with current concepts of neuroimmune interaction, in which inflammatory signaling, altered vagal modulation, and short-term blood pressure dysregulation can affect both cardiac and vascular autonomic pathways in a variety of post infection syndromes [7,8,10,12,13,14,36,44,47]. Experimental work on the “inflammatory reflex” further supports the notion that vagal activity may be modified during systemic infection, contributing to transient autonomic imbalance [57,58,59,60]. These findings show the importance of incorporating autonomic assessment into the clinical evaluation of patients with acute *C. burnetii* infection, particularly when symptoms are not easily explained by standard diagnostic testing. The results may also be informative for a wide range of clinicians including infectious disease specialists, neurologists, cardiologists, and general internal medicine physicians who encounter autonomic manifestations in the setting of systemic infection.

### Study Limitations

This study has several important limitations: (1) Sample size—We strongly recommend that future studies include a larger sample size and, if possible, a more precisely sex- and age-matched control group. Additionally, we suggest that future research adopt a longitudinal design to assess both the effect of time and potential therapeutic interventions on the observed changes. (2) Inclusion criteria—We propose that future studies utilize alternative serological methods, primarily those based on changes in IgG or IgM titers over a defined time period, as well as include patients with a more typical clinical presentation. (3) The use of additional diagnostic modalities—Future research should consider incorporating more comprehensive assessments of autonomic nervous system function, such as small fiber neuropathy evaluation, deceleration/acceleration capacity, heart rate turbulence, and other relevant tests.

## 5. Conclusions

This study demonstrated measurable autonomic dysfunction in patients presenting with polymorphic symptoms and positive IgM antibodies to *Coxiella burnetii*. Across complementary diagnostic modalities, the autonomic profile was characterized primarily by parasympathetic impairment, reduced baroreflex responsiveness, and distinct hemodynamic patterns during HUTT. Although causal relationships cannot be inferred from this cross-sectional design, the consistency of these findings across multiple test modalities suggests that autonomic involvement represents a relevant clinical feature in the setting of acute *Coxiella burnetii* infection. Further longitudinal studies are needed to clarify underlying mechanisms and to determine the long-term significance of these abnormalities. Taken together, our results highlight the importance of incorporating structured autonomic assessment in patients with acute Q fever who present with persistent, unexplained symptoms.

## Figures and Tables

**Table 1:** Demographic characteristics of study population.

	Coxiella Group N = 100	Control Group N = 56	*p* Value
Age (yrs.) (mean ± SD)	45 ± 15.3	41 ± 10.7	0.084 ^a^
Female (n, %)	80 (80%)	37 (66.1%)	0.054 ^b^
ME/CFS (n, %)	35 (35%)	/	
PCS (n, %)	5 (5%)	/	
Previous syncope (n, %)	59 (59%)	/	
HTA (n, %)	2 (2%)	/	
DM (n, %)	0	/	
GERD (n, %)	4 (4%)	/	
Pneumonia in the previous year (n, %)	3 (3%)	/	

Yrs.—Year; ME/CFS—Myalgic encephalomyelitis/Chronic Fatigue Syndrome; PCS—Post COVID Syndrome; HTA—Hypertension; DM—Diabetes Mellitus; GERD—Gastroesophageal reflux disease; ^a^—Independent Samples *t* test; ^b^—Pearson Chi Square.

**Table 2:** Results of Cardiovascular reflex tests of the study population.

	Coxiella Group N = 100	Control Group N = 56	*p* Value
HGT (n, %)	100 (100%)	40 (71.4%)	<0.001 ^a^
OH (n, %)	13 (13%)	0	0.004 ^b^
DS (n, %)	100 (100%)	40 (71.4%)	<0.001 ^a^
VM (n, %)	31 (31%)	15 (26.8%)	0.580 ^a^
HRB (n, %)	65 (65%)	10 (17.9%)	<0.001 ^a^
HRS (n, %)	96 (96%)	27 (48.2%)	<0.001 ^a^
Definite DP (n, %)	72 (72%)	10 (17.9%)	<0.001 ^a^
Score of AN (mead ± SD)	6.5 ± 1.5	4.3 ± 1.6	<0.001 ^c^

HGT—Hand grip test; OH—Orthostatic hypotension; DS—Sympathetic dysfunction; VM—Valsalva maneuver; HRB—Heart rate response to deep breathing; HRS—Heart rate response to standing; DP—Parasympathetic dysfunction; AN—autonomic neuropathy; SD—Standard deviation; ^a^—Pearson Chi Square; ^b^—Fisher Exact test; ^c^—Independent samples *t* test.

**Table 3:** Results of beat-to-beat analysis of the study population.

	Coxiella Group N = 100	Control Group N = 56	*p* Value
HR (bpm) (Mdn (IQR))	80.3 (58.9–96)	69.9 (63.8–75.4)	0.015 ^a^
SBP (mmHg) (mean ± SD)	110.2 ± 14.1	113.5 ± 11.7	0.399 ^b^
DBP (mmHg) (mean ± SD)	71.7 ± 9.2	75.8 ± 9.1	0.167 ^b^
LF (nu) (mean ± SD)	59.9 ± 16.8	60.6 ± 16.5	0.908 ^b^
PSD (ms^2^) (Mdn (IQR))	499 (249–2502)	1378 (649–3356)	0.106 ^b^
VLF (ms^2^) (Mdn (IQR))	117 (64–396)	300 (152–734)	0.058 ^b^
LF (ms^2^) (Mdn (IQR))	230 (128–450)	611 (195–1134)	0.039 ^b^
HF (ms^2^) (Mdn (IQR))	170 (33–445)	310 (122–736)	0.194 ^b^
LF/HF (nu) (Mdn (IQR))	1.5 (1.2–3)	2 (1–3)	0.641 ^b^
LF/HF (ms^2^) (Mdn (IQR))	1.2 (0.6–1.7)	1.4 (0.9–2.2)	0.415 ^b^
BRS (ms/mmHg) (Mdn (IQR))	11.4 (7–16.1)	15.3 (11.5–20.1)	0.080 ^b^
BEI (%) (Mdn (IQR))	76 (65.3–91.99)	116.5 (95.6–137.9)	0.003 ^b^

HR—Heart rate; bpm.—beats per minute; SBP—Systolic blood pressure; DBP—Diastolic blood pressure; mmHg—millimeters of mercury; LF—Low frequency component of HRV; nu—normalized units; PSD—Power spectral density; VLF—Very low frequency of HRV; HF—High frequency component of HRV; ms^2^—millisecond on square; BRS—Baroreflex sensitivity; ms/mmHg—milliseconds per millimeters of mercury; BEI—Baroreflex effectiveness index; Mdn—Median; IQR—25–75% interquartile range; SD—Standard deviation; ^a^—Mann–Whitney U test; ^b^—Independent samples *t* test.

**Table 4:** Parameters of 24 h Holter ECG monitoring of the study population.

	Coxiella Group N = 100	Control Group N = 56	*p* Value
HR (bpm) (mean ± SD)	77.2 ± 6.8	75.8 ± 7.5	0.421 ^a^
SDNN (ms) (mean ± SD)	145.2 ± 32.1	152.6 ± 36.4	0.397 ^a^
RMSSD (ms) (Mdn (IQR))	35 (31–58.5)	33.5 (25.8–46)	0.167 ^b^
PNN50 (%)(Mdn (IQR))	10 (3.5–16.5)	10 (5.5–18)	0.908 ^b^
TP (ms^2^) (Mdn (IQR))	2979 (2259.3–4008.3)	3676 (2778.7–5085.2)	0.106 ^b^
VLF (ms^2^) (Mdn (IQR))	2009.9 (1432.8–2553.6)	2436.5 (1741.5–3471.5)	0.058 ^b^
LF (ms^2^) (Mdn (IQR))	846.6 (452.2–1086.5)	878.8 (649.8–1137.8)	0.360 ^b^
HF (ms^2^) (Mdn (IQR))	264 (158.9–335.9)	265.1 (179–429.4)	0.194 ^b^
LF/HF (ms^2^) (Mdn (IQR))	3.4 (2.4–4.6)	2.9 (2.1–4.5)	0.641 ^b^

HR—Heart rate; bpm.—beats per minute; SDNN—Standard deviation of NN interval; RMSSD—Root mean square of successive differences; PNN50—percentage of successive NN intervals which differ > 50 ms; TP—Total power; ms^2^—milliseconds on square; VLF—Very low frequency of HRV; LF—Low frequency component of HRV; HF—High frequency component of HRV; SD—Standard deviation; Mdn—Median; IQR—25–75% interquartile range. ^a^—Independent samples *t* test; ^b^—Mann Whitney U test.

## Data Availability

The data are available upon reasonable request.
